# *trans*-Bis[1,3-bis­(2-methoxy­phen­yl)triazenido]dimethano­lcadmium(II)

**DOI:** 10.1107/S160053680900676X

**Published:** 2009-02-28

**Authors:** Mohammad Kazem Rofouei, Mohammad Reza Melardi, Hamid Reza Khalili Ghaydari, Mohammad Barkhi

**Affiliations:** a Faculty of Chemistry, Tarbiat Moallem University, Tehran, Iran; b Department of Chemistry, Islamic Azad University, Karaj Branch, Karaj, Iran

## Abstract

In the title compound, [Cd(C_14_H_14_N_3_O_2_)_2_(CH_3_OH)_2_], each cadmium(II) center is six-coordinated by an N atom and an O atom of two 1,3-bis­(2-methoxy­phen­yl)triazene ligands and by the O atoms of two methanol mol­ecules. The distorted octa­hedral coordination geometry of the Cd atom has two N and two O atoms in the equatorial plane, and two O atoms in axial positions. The complex is stabilized by intra­molecular O—H⋯O and O—H⋯N hydrogen bonds. In the crystal structure the complexes are linked into chains *via* inter­molecular C—H⋯π stacking inter­actions. One of the methanol C atoms is disordered with ouccupancies of 0.7:0.3.

## Related literature

For complexes of the title ligand, see: Payehghadr *et al.* (2006 ▶); Rofouei, Shamsipur *et al.* (2006 ▶); Rofouei, Melardi *et al.* (2008 ▶); Rofouei & Hashempur (2008 ▶).
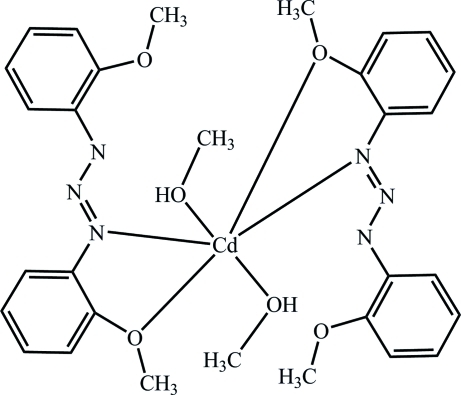


## Experimental

### 

#### Crystal data

[Cd(C_14_H_14_N_3_O_2_)_2_(CH_4_O)_2_]
*M* *_r_* = 689.05
Orthorhombic, 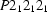

*a* = 11.0333 (10) Å
*b* = 13.1892 (12) Å
*c* = 21.4784 (17) Å
*V* = 3125.5 (5) Å^3^
*Z* = 4
Mo *K*α radiation
μ = 0.75 mm^−1^
*T* = 100 K
0.50 × 0.40 × 0.30 mm

#### Data collection

Bruker SMART APEXII CCD area-detector diffractometer
Absorption correction: multi-scan (*SADABS*; Sheldrick, 1996 ▶) *T*_min_ = 0.693, *T*_max_ = 0.806
40482 measured reflections
9074 independent reflections
8503 reflections with *I* > 2σ(*I*)
*R*_int_ = 0.041

#### Refinement

*R*[*F* ^2^ > 2σ(*F* ^2^)] = 0.055
*wR*(*F* ^2^) = 0.132
*S* = 1.07
9074 reflections
394 parameters
2 restraints
H-atom parameters constrained
Δρ_max_ = 2.65 e Å^−3^
Δρ_min_ = −2.09 e Å^−3^
Absolute structure: Flack (1983 ▶), 4009 Friedel pairs
Flack parameter: 0.04 (3)

### 

Data collection: *APEX2* (Bruker, 2007 ▶); cell refinement: *SAINT* (Bruker, 2007 ▶); data reduction: *SAINT*; program(s) used to solve structure: *SHELXS97* (Sheldrick, 2008 ▶); program(s) used to refine structure: *SHELXL97* (Sheldrick, 2008 ▶); molecular graphics: *SHELXTL* (Sheldrick, 2008 ▶); software used to prepare material for publication: *SHELXTL*.

## Supplementary Material

Crystal structure: contains datablocks I, global. DOI: 10.1107/S160053680900676X/su2094sup1.cif

Structure factors: contains datablocks I. DOI: 10.1107/S160053680900676X/su2094Isup2.hkl

Additional supplementary materials:  crystallographic information; 3D view; checkCIF report

## Figures and Tables

**Table 1:** Hydrogen-bond geometry (Å, °)

*D*—H⋯*A*	*D*—H	H⋯*A*	*D*⋯*A*	*D*—H⋯*A*
O5—H5*O*⋯N3	0.91	1.88	2.710 (6)	150
O6—H6*O*⋯O4	0.86	1.92	2.739 (5)	157
C23—H23*A*⋯*Cg*1^i^	0.95	2.82	3.655 (6)	147

Symmetry code: (i) 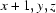. *Cg*1 is the centroid of the C1–C6 ring.

**Fig. 1.**
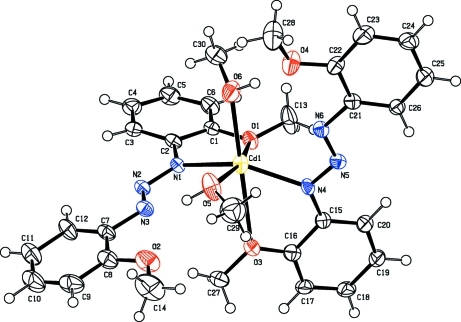
The molecular structure of the title compound with thermal ellipsoids drawn at the 50% probability level.

**Fig. 2.**
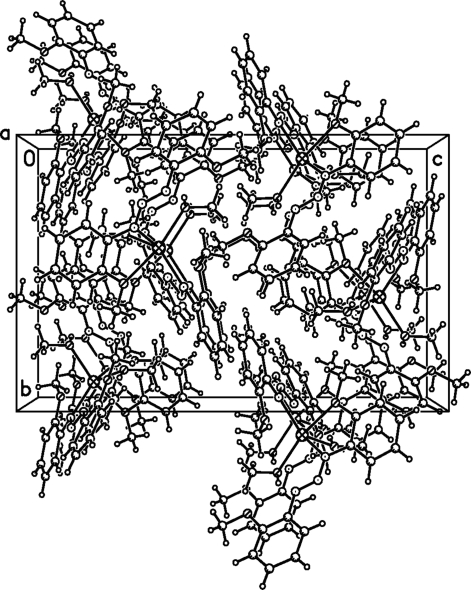
A view along the a axis of the crystal packing of the title compound.

**Fig. 3.**
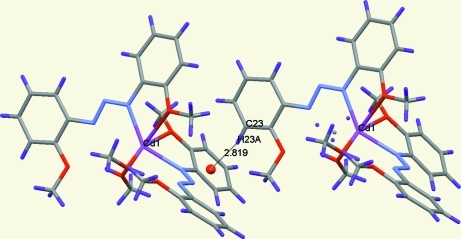
C—H···π stacking interactions in the title compound (see Table 1 for details).

## supplementary crystallographic information

### Comment

Recently, we have reported on the crystal structures of silver(I), copper(I), and mercry(II) complexes of the ligand [1,3-di(2-methoxy)benzene]triazene (Payehghadr *et al.*, 2006; Rofouei & Hashempur 2008; Rofouei, Melardi *et al.*, 2008). Here we report on the crystal structure of the cadmium(II) complex of the same ligand.

The molecular structure of the title complex is illustrated in Fig. 1. A view along the a axis of the crystal packing is given in Fig. 2. The title complex crystallizes in the non-centrosymmetric space group P2_1_2_1_2_1_; the Flack X factor is 0.04 (3). Each cadmium(II) center is six-coordinated by an N-atom and an O-atom of two [1,3-di(2-methoxy)benzene]triazene ligands, and by the O-atom of two molecules of methanol. The octahedral coordination geometry of the cadmium atom has two N- and two O-atoms in the equatorial plane, and two O-atoms in axial positions. The Cd—N and Cd—O bond lengths are in the expected range for such coordination bonds.

The complex is stabilized by intramolecular O—H···O and O—H···N hydrogen bonds (Table 1). Another noticeable feature of the title compound is the presence of C—H···π stacking interactions (Fig. 3 and Table 1).

### Experimental

The synthesis of the ligand 1,3-bis(2-methoxybenzene)triazene has been reported previously (Rofouei, Shamsipur *et al.*, 2006). For the preparation of the cadmium(II) complex a solution of cadmium acetate monohydrate (248 mg, 1 mmol) in methanol (10 ml) was carefully added to a solution of 1,3-bis(2-methoxybenzene)triazene, (514 mg, 2 mmol) in dichloromethane (20 ml) with stirring at 40 °C. The solution was then cooled to rt and after several days needle-like red crystals of the title compound were isolated (yield; 550 mg, 80%).

### Refinement

The OH H-atoms could be located in difference Fourier syntheses, and were refined as riding: O-H = 0.86 - 0.91 Å with U_iso_(H) = 1.5U_eq_(parent O-atom). The C-bound H-atoms were included in calculated positions and treated as riding: C-H = 0.95 - 0.98 Å with U_iso_(H) = 1.2 or 1.5U_eq_(parent C-atom).

### Crystal data

**Table:**

[Cd(C_14_H_14_N_3_O_2_)_2_(CH_4_O)_2_]	*F*(000) = 1416
*M**_r_* = 689.05	*D*_x_ = 1.464 Mg m^−^^3^
Orthorhombic, *P*2_1_2_1_2_1_	Mo *K*α radiation, λ = 0.71073 Å
Hall symbol: P 2ac 2ab	Cell parameters from 6454 reflections
*a* = 11.0333 (10) Å	θ = 2.4–28.4°
*b* = 13.1892 (12) Å	µ = 0.75 mm^−^^1^
*c* = 21.4784 (17) Å	*T* = 100 K
*V* = 3125.5 (5) Å^3^	Prism, red
*Z* = 4	0.50 × 0.40 × 0.30 mm

### Data collection

**Table:**

Bruker SMART APEXII CCD area-detector diffractometer	9074 independent reflections
Radiation source: fine-focus sealed tube	8503 reflections with *I* > 2σ(*I*)
graphite	*R*_int_ = 0.041
ω scans	θ_max_ = 30.0°, θ_min_ = 1.8°
Absorption correction: multi-scan (*SADABS*; Sheldrick, 1996)	*h* = −15→15
*T*_min_ = 0.693, *T*_max_ = 0.806	*k* = −18→18
40482 measured reflections	*l* = −30→30

### Refinement

**Table:**

Refinement on *F*^2^	Secondary atom site location: difference Fourier map
Least-squares matrix: full	Hydrogen site location: difference Fourier map
*R*[*F*^2^ > 2σ(*F*^2^)] = 0.055	H-atom parameters constrained
*wR*(*F*^2^) = 0.132	*w* = 1/[σ^2^(*F*_o_^2^) + (0.0582*P*)^2^ + 7.7532*P*] where *P* = (*F*_o_^2^ + 2*F*_c_^2^)/3
*S* = 1.07	(Δ/σ)_max_ = 0.001
9074 reflections	Δρ_max_ = 2.65 e Å^−^^3^
394 parameters	Δρ_min_ = −2.09 e Å^−^^3^
2 restraints	Absolute structure: Flack (1983), 4009 Friedel pairs
Primary atom site location: structure-invariant direct methods	Flack parameter: 0.04 (3)

### Special details

**Table:**

Geometry. All e.s.d.'s (except the e.s.d. in the dihedral angle between two l.s. planes) are estimated using the full covariance matrix. The cell e.s.d.'s are taken into account individually in the estimation of e.s.d.'s in distances, angles and torsion angles; correlations between e.s.d.'s in cell parameters are only used when they are defined by crystal symmetry. An approximate (isotropic) treatment of cell e.s.d.'s is used for estimating e.s.d.'s involving l.s. planes.
Refinement. Refinement of *F*^2^ against ALL reflections. The weighted *R*-factor *wR* and goodness of fit *S* are based on *F*^2^, conventional *R*-factors *R* are based on *F*, with *F* set to zero for negative *F*^2^. The threshold expression of *F*^2^ > σ(*F*^2^) is used only for calculating *R*-factors(gt) *etc*. and is not relevant to the choice of reflections for refinement. *R*-factors based on *F*^2^ are statistically about twice as large as those based on *F*, and *R*- factors based on ALL data will be even larger.

### Fractional atomic coordinates and isotropic or equivalent isotropic displacement parameters (Å^2^)

**Table:**

	*x*	*y*	*z*	*U*_iso_*/*U*_eq_	Occ. (<1)
Cd1	0.51781 (3)	0.91405 (2)	0.161135 (15)	0.02659 (8)	
O1	0.4292 (3)	1.0353 (2)	0.23583 (15)	0.0268 (6)	
O2	0.3751 (4)	0.6433 (4)	0.03403 (18)	0.0480 (10)	
O3	0.3595 (3)	0.9780 (3)	0.07339 (16)	0.0324 (7)	
O4	0.8882 (3)	0.8905 (3)	0.2077 (2)	0.0440 (10)	
O5	0.5567 (4)	0.7740 (3)	0.1042 (2)	0.0455 (10)	
H5O	0.4832	0.7428	0.1054	0.068*	
O6	0.6498 (3)	0.8632 (4)	0.2391 (2)	0.0489 (11)	
H6O	0.7195	0.8896	0.2305	0.073*	
N1	0.3595 (3)	0.8468 (3)	0.21076 (16)	0.0205 (6)	
N2	0.2922 (3)	0.7694 (3)	0.19269 (17)	0.0232 (7)	
N3	0.3298 (3)	0.7260 (3)	0.14246 (17)	0.0257 (7)	
N4	0.5715 (3)	1.0479 (3)	0.10768 (17)	0.0229 (7)	
N5	0.6843 (3)	1.0783 (3)	0.11376 (15)	0.0234 (6)	
N6	0.7468 (3)	1.0152 (3)	0.14575 (17)	0.0234 (7)	
C1	0.3554 (4)	0.9904 (3)	0.28023 (19)	0.0222 (7)	
C2	0.3200 (4)	0.8898 (3)	0.2670 (2)	0.0231 (8)	
C3	0.2489 (4)	0.8391 (4)	0.3104 (2)	0.0270 (8)	
H3A	0.2279	0.7701	0.3036	0.032*	
C4	0.2081 (5)	0.8886 (4)	0.3639 (2)	0.0337 (10)	
H4A	0.1579	0.8537	0.3928	0.040*	
C5	0.2404 (5)	0.9878 (4)	0.3750 (2)	0.0361 (11)	
H5A	0.2107	1.0215	0.4109	0.043*	
C6	0.3162 (4)	1.0390 (4)	0.3338 (2)	0.0320 (9)	
H6A	0.3407	1.1067	0.3422	0.038*	
C7	0.2556 (4)	0.6430 (3)	0.1242 (2)	0.0277 (9)	
C8	0.2825 (5)	0.5977 (4)	0.0674 (2)	0.0376 (11)	
C9	0.2153 (6)	0.5154 (5)	0.0456 (3)	0.0469 (14)	
H9A	0.2344	0.4852	0.0066	0.056*	
C10	0.1206 (6)	0.4780 (5)	0.0811 (3)	0.0523 (16)	
H10A	0.0740	0.4226	0.0661	0.063*	
C11	0.0932 (6)	0.5205 (4)	0.1384 (3)	0.0455 (13)	
H11A	0.0286	0.4946	0.1629	0.055*	
C12	0.1629 (4)	0.6025 (3)	0.1594 (3)	0.0352 (10)	
H12A	0.1459	0.6309	0.1991	0.042*	
C13	0.4891 (7)	1.1271 (4)	0.2534 (3)	0.0533 (17)	
H13A	0.4285	1.1796	0.2619	0.080*	
H13B	0.5420	1.1494	0.2194	0.080*	
H13C	0.5377	1.1151	0.2908	0.080*	
C14	0.4088 (7)	0.5967 (7)	−0.0236 (3)	0.069 (2)	
H14A	0.4676	0.6398	−0.0452	0.104*	
H14B	0.3367	0.5881	−0.0497	0.104*	
H14C	0.4452	0.5303	−0.0153	0.104*	
C15	0.4934 (4)	1.1159 (3)	0.07856 (18)	0.0234 (8)	
C16	0.3771 (4)	1.0781 (4)	0.06114 (19)	0.0276 (8)	
C17	0.2928 (4)	1.1424 (4)	0.0334 (2)	0.0309 (9)	
H17A	0.2156	1.1169	0.0218	0.037*	
C18	0.3205 (4)	1.2424 (4)	0.0228 (2)	0.0330 (10)	
H18A	0.2620	1.2854	0.0039	0.040*	
C19	0.4324 (4)	1.2816 (4)	0.0393 (2)	0.0328 (10)	
H19A	0.4511	1.3508	0.0316	0.039*	
C20	0.5178 (4)	1.2176 (3)	0.06748 (19)	0.0273 (8)	
H20A	0.5943	1.2445	0.0793	0.033*	
C21	0.8696 (3)	1.0449 (3)	0.1529 (2)	0.0230 (8)	
C22	0.9429 (4)	0.9784 (4)	0.1876 (2)	0.0287 (9)	
C23	1.0637 (4)	1.0021 (5)	0.1995 (3)	0.0418 (13)	
H23A	1.1120	0.9581	0.2242	0.050*	
C24	1.1133 (4)	1.0911 (5)	0.1749 (2)	0.0365 (10)	
H24A	1.1956	1.1078	0.1830	0.044*	
C25	1.0437 (4)	1.1540 (4)	0.1393 (2)	0.0328 (10)	
H25A	1.0783	1.2136	0.1220	0.039*	
C26	0.9219 (4)	1.1314 (3)	0.1281 (2)	0.0250 (8)	
H26A	0.8745	1.1757	0.1031	0.030*	
C27	0.2458 (5)	0.9342 (5)	0.0562 (2)	0.0404 (12)	
H27A	0.2482	0.8608	0.0635	0.061*	
H27B	0.2301	0.9473	0.0121	0.061*	
H27C	0.1810	0.9644	0.0814	0.061*	
C28	0.9622 (5)	0.8102 (5)	0.2272 (4)	0.0603 (19)	
H28A	0.9137	0.7482	0.2305	0.090*	
H28B	0.9972	0.8264	0.2680	0.090*	
H28C	1.0275	0.8000	0.1969	0.090*	
C29	0.6093 (11)	0.7716 (10)	0.0456 (4)	0.064 (3)*	0.70
H29A	0.6073	0.7021	0.0295	0.096*	0.70
H29B	0.6935	0.7946	0.0485	0.096*	0.70
H29C	0.5642	0.8163	0.0175	0.096*	0.70
C29'	0.566 (3)	0.808 (3)	0.0417 (7)	0.077 (8)*	0.30
H29D	0.5572	0.7501	0.0134	0.116*	0.30
H29E	0.6451	0.8398	0.0352	0.116*	0.30
H29F	0.5017	0.8574	0.0333	0.116*	0.30
C30	0.6405 (5)	0.8652 (5)	0.3049 (3)	0.0467 (13)	
H30A	0.7028	0.8212	0.3230	0.070*	
H30B	0.5601	0.8411	0.3175	0.070*	
H30C	0.6522	0.9348	0.3198	0.070*	

### Atomic displacement parameters (Å^2^)

**Table:**

	*U*^11^	*U*^22^	*U*^33^	*U*^12^	*U*^13^	*U*^23^
Cd1	0.02329 (12)	0.02162 (12)	0.03485 (14)	0.00110 (11)	0.00678 (12)	−0.00016 (12)
O1	0.0261 (15)	0.0220 (14)	0.0323 (16)	−0.0034 (12)	0.0021 (12)	−0.0064 (12)
O2	0.048 (2)	0.061 (3)	0.0348 (19)	−0.006 (2)	0.0054 (17)	−0.0208 (18)
O3	0.0271 (16)	0.0336 (18)	0.0366 (18)	−0.0081 (13)	−0.0058 (14)	0.0037 (14)
O4	0.0236 (16)	0.038 (2)	0.070 (3)	0.0085 (14)	0.0058 (16)	0.0222 (18)
O5	0.0288 (18)	0.0344 (19)	0.073 (3)	−0.0035 (15)	0.0166 (18)	−0.0148 (19)
O6	0.0204 (17)	0.061 (3)	0.065 (3)	−0.0055 (17)	−0.0038 (17)	0.032 (2)
N1	0.0189 (15)	0.0203 (15)	0.0224 (15)	−0.0018 (12)	−0.0004 (12)	−0.0030 (12)
N2	0.0208 (16)	0.0201 (16)	0.0285 (17)	0.0002 (13)	−0.0048 (13)	−0.0031 (13)
N3	0.0211 (16)	0.0265 (18)	0.0294 (17)	0.0019 (13)	−0.0018 (13)	−0.0039 (14)
N4	0.0150 (15)	0.0250 (16)	0.0288 (17)	−0.0040 (12)	0.0019 (13)	0.0020 (13)
N5	0.0177 (14)	0.0298 (17)	0.0227 (15)	0.0007 (14)	0.0019 (11)	−0.0016 (15)
N6	0.0159 (14)	0.0253 (17)	0.0291 (18)	0.0024 (12)	0.0017 (12)	−0.0001 (13)
C1	0.0201 (18)	0.0230 (19)	0.0234 (18)	0.0004 (14)	−0.0040 (14)	−0.0028 (15)
C2	0.0174 (17)	0.026 (2)	0.0260 (19)	0.0011 (14)	−0.0030 (14)	−0.0051 (15)
C3	0.0225 (19)	0.030 (2)	0.028 (2)	0.0003 (16)	−0.0046 (16)	0.0017 (17)
C4	0.033 (2)	0.045 (3)	0.0239 (19)	−0.0050 (19)	0.0011 (17)	−0.0019 (18)
C5	0.040 (3)	0.039 (3)	0.029 (2)	0.000 (2)	0.0004 (19)	−0.012 (2)
C6	0.029 (2)	0.033 (2)	0.034 (2)	−0.0015 (16)	−0.0003 (19)	−0.009 (2)
C7	0.027 (2)	0.0216 (19)	0.034 (2)	0.0015 (15)	−0.0083 (17)	−0.0071 (16)
C8	0.039 (2)	0.035 (3)	0.039 (2)	−0.001 (2)	−0.0045 (19)	−0.010 (2)
C9	0.058 (4)	0.040 (3)	0.042 (3)	0.005 (3)	−0.014 (3)	−0.017 (2)
C10	0.058 (4)	0.038 (3)	0.060 (4)	−0.008 (3)	−0.014 (3)	−0.019 (3)
C11	0.040 (3)	0.037 (3)	0.059 (3)	−0.008 (2)	−0.007 (2)	−0.010 (2)
C12	0.035 (2)	0.023 (2)	0.048 (3)	−0.0033 (15)	−0.003 (2)	−0.015 (2)
C13	0.067 (4)	0.039 (3)	0.054 (3)	−0.027 (3)	0.022 (3)	−0.019 (2)
C14	0.071 (5)	0.088 (6)	0.049 (3)	−0.009 (4)	0.011 (3)	−0.039 (4)
C15	0.0172 (19)	0.0297 (18)	0.0232 (16)	0.0001 (14)	0.0050 (14)	0.0005 (13)
C16	0.0228 (18)	0.034 (2)	0.0262 (18)	0.0000 (18)	0.0023 (14)	−0.0011 (19)
C17	0.023 (2)	0.044 (3)	0.025 (2)	−0.0029 (18)	−0.0042 (16)	0.0062 (18)
C18	0.022 (2)	0.043 (3)	0.034 (2)	0.0020 (19)	−0.0067 (17)	0.010 (2)
C19	0.028 (2)	0.034 (2)	0.036 (2)	−0.0033 (18)	−0.0043 (18)	0.0115 (19)
C20	0.0176 (16)	0.033 (2)	0.0311 (19)	0.0018 (17)	0.0027 (16)	0.0057 (16)
C21	0.0144 (15)	0.0309 (19)	0.0237 (19)	0.0040 (14)	0.0040 (14)	−0.0017 (15)
C22	0.0186 (18)	0.034 (2)	0.034 (2)	0.0083 (16)	0.0056 (16)	0.0106 (18)
C23	0.017 (2)	0.062 (4)	0.047 (3)	0.008 (2)	−0.0005 (19)	0.019 (3)
C24	0.0221 (19)	0.051 (3)	0.037 (2)	0.000 (2)	−0.0034 (15)	0.005 (2)
C25	0.019 (2)	0.041 (3)	0.038 (2)	0.0002 (17)	0.0052 (16)	0.0045 (19)
C26	0.024 (2)	0.027 (2)	0.0243 (19)	0.0000 (16)	0.0000 (15)	0.0013 (15)
C27	0.036 (2)	0.053 (4)	0.033 (2)	−0.013 (2)	−0.0098 (19)	0.003 (2)
C28	0.029 (3)	0.044 (3)	0.107 (6)	0.008 (2)	0.004 (3)	0.025 (3)
C30	0.031 (3)	0.056 (4)	0.052 (3)	−0.004 (2)	−0.008 (2)	0.003 (3)

### Geometric parameters (Å, °)

**Table:**

Cd1—N4	2.188 (4)	C11—C12	1.402 (7)
Cd1—N1	2.230 (3)	C11—H11A	0.9500
Cd1—O5	2.257 (4)	C12—H12A	0.9500
Cd1—O6	2.318 (4)	C13—H13A	0.9800
Cd1—O1	2.467 (3)	C13—H13B	0.9800
Cd1—O3	2.704 (3)	C13—H13C	0.9800
O1—C1	1.387 (5)	C14—H14A	0.9800
O1—C13	1.430 (6)	C14—H14B	0.9800
O2—C8	1.385 (7)	C14—H14C	0.9800
O2—C14	1.430 (7)	C15—C20	1.389 (6)
O3—C16	1.360 (6)	C15—C16	1.426 (6)
O3—C27	1.430 (6)	C16—C17	1.393 (7)
O4—C22	1.377 (6)	C17—C18	1.373 (7)
O4—C28	1.401 (6)	C17—H17A	0.9500
O5—C29	1.386 (8)	C18—C19	1.385 (7)
O5—C29'	1.419 (10)	C18—H18A	0.9500
O5—H5O	0.91	C19—C20	1.402 (6)
O6—C30	1.418 (8)	C19—H19A	0.9500
O6—H6O	0.86	C20—H20A	0.9500
N1—N2	1.321 (5)	C21—C26	1.385 (6)
N1—C2	1.404 (5)	C21—C22	1.406 (6)
N2—N3	1.289 (5)	C22—C23	1.392 (7)
N3—C7	1.423 (6)	C23—C24	1.399 (8)
N4—N5	1.313 (5)	C23—H23A	0.9500
N4—C15	1.392 (5)	C24—C25	1.365 (7)
N5—N6	1.281 (5)	C24—H24A	0.9500
N6—C21	1.419 (5)	C25—C26	1.397 (6)
C1—C6	1.386 (6)	C25—H25A	0.9500
C1—C2	1.412 (6)	C26—H26A	0.9500
C2—C3	1.390 (6)	C27—H27A	0.9800
C3—C4	1.397 (7)	C27—H27B	0.9800
C3—H3A	0.9500	C27—H27C	0.9800
C4—C5	1.376 (8)	C28—H28A	0.9800
C4—H4A	0.9500	C28—H28B	0.9800
C5—C6	1.393 (7)	C28—H28C	0.9800
C5—H5A	0.9500	C29—H29A	0.9800
C6—H6A	0.9500	C29—H29B	0.9800
C7—C12	1.379 (7)	C29—H29C	0.9800
C7—C8	1.391 (7)	C29'—H29D	0.9800
C8—C9	1.396 (8)	C29'—H29E	0.9800
C9—C10	1.385 (10)	C29'—H29F	0.9800
C9—H9A	0.9500	C30—H30A	0.9800
C10—C11	1.385 (9)	C30—H30B	0.9800
C10—H10A	0.9500	C30—H30C	0.9800
			
N4—Cd1—N1	141.63 (13)	C11—C12—H12A	119.0
N4—Cd1—O5	108.95 (13)	O1—C13—H13A	109.5
N1—Cd1—O5	94.72 (13)	O1—C13—H13B	109.5
N4—Cd1—O6	116.27 (14)	H13A—C13—H13B	109.5
N1—Cd1—O6	91.80 (13)	O1—C13—H13C	109.5
O5—Cd1—O6	92.01 (18)	H13A—C13—H13C	109.5
N4—Cd1—O1	85.74 (12)	H13B—C13—H13C	109.5
N1—Cd1—O1	68.68 (12)	O2—C14—H14A	109.5
O5—Cd1—O1	163.40 (12)	O2—C14—H14B	109.5
O6—Cd1—O1	88.09 (15)	H14A—C14—H14B	109.5
N4—Cd1—O3	63.74 (12)	O2—C14—H14C	109.5
N1—Cd1—O3	87.22 (12)	H14A—C14—H14C	109.5
O5—Cd1—O3	90.02 (14)	H14B—C14—H14C	109.5
O6—Cd1—O3	177.81 (15)	C20—C15—N4	125.4 (4)
O1—Cd1—O3	89.73 (11)	C20—C15—C16	117.8 (4)
C1—O1—C13	116.8 (4)	N4—C15—C16	116.7 (4)
C1—O1—Cd1	113.8 (2)	O3—C16—C17	125.4 (4)
C13—O1—Cd1	122.5 (3)	O3—C16—C15	114.7 (4)
C8—O2—C14	116.9 (5)	C17—C16—C15	120.0 (4)
C16—O3—C27	117.9 (4)	C18—C17—C16	120.5 (4)
C16—O3—Cd1	110.2 (2)	C18—C17—H17A	119.7
C27—O3—Cd1	128.3 (3)	C16—C17—H17A	119.7
C22—O4—C28	118.4 (4)	C17—C18—C19	121.0 (4)
C29—O5—Cd1	126.2 (6)	C17—C18—H18A	119.5
C29'—O5—Cd1	105.5 (15)	C19—C18—H18A	119.5
C29—O5—H5O	112.8	C18—C19—C20	119.0 (4)
C29'—O5—H5O	103.4	C18—C19—H19A	120.5
Cd1—O5—H5O	100.7	C20—C19—H19A	120.5
C30—O6—Cd1	132.0 (4)	C15—C20—C19	121.7 (4)
C30—O6—H6O	105.7	C15—C20—H20A	119.2
Cd1—O6—H6O	106.7	C19—C20—H20A	119.2
N2—N1—C2	113.0 (3)	C26—C21—C22	118.5 (4)
N2—N1—Cd1	127.4 (3)	C26—C21—N6	125.7 (4)
C2—N1—Cd1	119.6 (3)	C22—C21—N6	115.7 (4)
N3—N2—N1	114.1 (4)	O4—C22—C23	123.4 (4)
N2—N3—C7	112.7 (4)	O4—C22—C21	116.1 (4)
N5—N4—C15	115.7 (4)	C23—C22—C21	120.5 (5)
N5—N4—Cd1	116.8 (3)	C22—C23—C24	119.6 (5)
C15—N4—Cd1	126.0 (3)	C22—C23—H23A	120.2
N6—N5—N4	111.4 (4)	C24—C23—H23A	120.2
N5—N6—C21	113.1 (4)	C25—C24—C23	120.1 (4)
C6—C1—O1	123.8 (4)	C25—C24—H24A	120.0
C6—C1—C2	121.0 (4)	C23—C24—H24A	120.0
O1—C1—C2	115.2 (4)	C24—C25—C26	120.5 (5)
C3—C2—N1	123.9 (4)	C24—C25—H25A	119.7
C3—C2—C1	118.3 (4)	C26—C25—H25A	119.7
N1—C2—C1	117.8 (4)	C21—C26—C25	120.7 (4)
C2—C3—C4	120.5 (4)	C21—C26—H26A	119.6
C2—C3—H3A	119.7	C25—C26—H26A	119.6
C4—C3—H3A	119.7	O3—C27—H27A	109.5
C5—C4—C3	120.2 (5)	O3—C27—H27B	109.5
C5—C4—H4A	119.9	H27A—C27—H27B	109.5
C3—C4—H4A	119.9	O3—C27—H27C	109.5
C4—C5—C6	120.5 (5)	H27A—C27—H27C	109.5
C4—C5—H5A	119.8	H27B—C27—H27C	109.5
C6—C5—H5A	119.8	O4—C28—H28A	109.5
C1—C6—C5	119.3 (4)	O4—C28—H28B	109.5
C1—C6—H6A	120.3	H28A—C28—H28B	109.5
C5—C6—H6A	120.3	O4—C28—H28C	109.5
C12—C7—C8	118.2 (4)	H28A—C28—H28C	109.5
C12—C7—N3	125.0 (4)	H28B—C28—H28C	109.5
C8—C7—N3	116.7 (4)	O5—C29—H29A	109.5
O2—C8—C7	115.1 (5)	O5—C29—H29B	109.5
O2—C8—C9	123.8 (5)	O5—C29—H29C	109.5
C7—C8—C9	121.0 (5)	O5—C29'—H29D	109.5
C10—C9—C8	119.5 (5)	O5—C29'—H29E	109.5
C10—C9—H9A	120.2	H29D—C29'—H29E	109.5
C8—C9—H9A	120.2	O5—C29'—H29F	109.5
C11—C10—C9	120.7 (5)	H29D—C29'—H29F	109.5
C11—C10—H10A	119.7	H29E—C29'—H29F	109.5
C9—C10—H10A	119.7	O6—C30—H30A	109.5
C10—C11—C12	118.6 (6)	O6—C30—H30B	109.5
C10—C11—H11A	120.7	H30A—C30—H30B	109.5
C12—C11—H11A	120.7	O6—C30—H30C	109.5
C7—C12—C11	121.9 (5)	H30A—C30—H30C	109.5
C7—C12—H12A	119.0	H30B—C30—H30C	109.5
			
N4—Cd1—O1—C1	168.6 (3)	Cd1—N1—C2—C3	−160.1 (3)
N1—Cd1—O1—C1	17.8 (3)	N2—N1—C2—C1	−158.8 (4)
O5—Cd1—O1—C1	15.7 (7)	Cd1—N1—C2—C1	19.5 (5)
O6—Cd1—O1—C1	−74.9 (3)	C6—C1—C2—C3	−2.7 (6)
O3—Cd1—O1—C1	104.9 (3)	O1—C1—C2—C3	178.1 (4)
N4—Cd1—O1—C13	−41.5 (4)	C6—C1—C2—N1	177.6 (4)
N1—Cd1—O1—C13	167.7 (4)	O1—C1—C2—N1	−1.5 (5)
O5—Cd1—O1—C13	165.7 (6)	N1—C2—C3—C4	−176.8 (4)
O6—Cd1—O1—C13	75.1 (4)	C1—C2—C3—C4	3.6 (6)
O3—Cd1—O1—C13	−105.1 (4)	C2—C3—C4—C5	−1.5 (7)
N4—Cd1—O3—C16	−23.7 (3)	C3—C4—C5—C6	−1.5 (8)
N1—Cd1—O3—C16	130.4 (3)	O1—C1—C6—C5	178.9 (4)
O5—Cd1—O3—C16	−134.9 (3)	C2—C1—C6—C5	−0.2 (7)
O1—Cd1—O3—C16	61.7 (3)	C4—C5—C6—C1	2.3 (8)
N4—Cd1—O3—C27	178.4 (4)	N2—N3—C7—C12	−8.4 (6)
N1—Cd1—O3—C27	−27.5 (4)	N2—N3—C7—C8	174.1 (4)
O5—Cd1—O3—C27	67.2 (4)	C14—O2—C8—C7	177.7 (6)
O1—Cd1—O3—C27	−96.2 (4)	C14—O2—C8—C9	−5.4 (9)
N4—Cd1—O5—C29	1.8 (8)	C12—C7—C8—O2	179.0 (5)
N1—Cd1—O5—C29	151.1 (7)	N3—C7—C8—O2	−3.3 (7)
O6—Cd1—O5—C29	−116.9 (7)	C12—C7—C8—C9	2.0 (8)
O1—Cd1—O5—C29	153.0 (8)	N3—C7—C8—C9	179.6 (5)
O3—Cd1—O5—C29	63.9 (7)	O2—C8—C9—C10	−177.0 (6)
N4—Cd1—O5—C29'	−19.7 (16)	C7—C8—C9—C10	−0.2 (9)
N1—Cd1—O5—C29'	129.6 (16)	C8—C9—C10—C11	−1.0 (10)
O6—Cd1—O5—C29'	−138.4 (16)	C9—C10—C11—C12	0.3 (10)
O1—Cd1—O5—C29'	131.5 (16)	C8—C7—C12—C11	−2.6 (8)
O3—Cd1—O5—C29'	42.4 (16)	N3—C7—C12—C11	180.0 (5)
N4—Cd1—O6—C30	110.3 (5)	C10—C11—C12—C7	1.5 (9)
N1—Cd1—O6—C30	−42.6 (5)	N5—N4—C15—C20	−13.8 (6)
O5—Cd1—O6—C30	−137.4 (5)	Cd1—N4—C15—C20	151.8 (3)
O1—Cd1—O6—C30	26.0 (5)	N5—N4—C15—C16	168.8 (4)
N4—Cd1—N1—N2	107.2 (4)	Cd1—N4—C15—C16	−25.5 (5)
O5—Cd1—N1—N2	−21.8 (4)	C27—O3—C16—C17	0.5 (7)
O6—Cd1—N1—N2	−113.9 (4)	Cd1—O3—C16—C17	−160.0 (4)
O1—Cd1—N1—N2	158.8 (4)	C27—O3—C16—C15	−179.0 (4)
O3—Cd1—N1—N2	68.0 (3)	Cd1—O3—C16—C15	20.6 (4)
N4—Cd1—N1—C2	−70.8 (4)	C20—C15—C16—O3	−179.9 (4)
O5—Cd1—N1—C2	160.2 (3)	N4—C15—C16—O3	−2.4 (5)
O6—Cd1—N1—C2	68.0 (3)	C20—C15—C16—C17	0.6 (6)
O1—Cd1—N1—C2	−19.2 (3)	N4—C15—C16—C17	178.1 (4)
O3—Cd1—N1—C2	−110.0 (3)	O3—C16—C17—C18	−179.6 (4)
C2—N1—N2—N3	−177.3 (3)	C15—C16—C17—C18	−0.2 (7)
Cd1—N1—N2—N3	4.6 (5)	C16—C17—C18—C19	0.0 (8)
N1—N2—N3—C7	−180.0 (4)	C17—C18—C19—C20	−0.3 (8)
N1—Cd1—N4—N5	146.5 (3)	N4—C15—C20—C19	−178.2 (4)
O5—Cd1—N4—N5	−88.5 (3)	C16—C15—C20—C19	−0.9 (6)
O6—Cd1—N4—N5	13.7 (4)	C18—C19—C20—C15	0.8 (7)
O1—Cd1—N4—N5	99.4 (3)	N5—N6—C21—C26	−1.1 (6)
O3—Cd1—N4—N5	−168.7 (3)	N5—N6—C21—C22	−179.9 (4)
N1—Cd1—N4—C15	−19.0 (5)	C28—O4—C22—C23	−16.3 (9)
O5—Cd1—N4—C15	106.0 (3)	C28—O4—C22—C21	163.1 (6)
O6—Cd1—N4—C15	−151.9 (3)	C26—C21—C22—O4	−175.8 (4)
O1—Cd1—N4—C15	−66.1 (3)	N6—C21—C22—O4	3.1 (6)
O3—Cd1—N4—C15	25.7 (3)	C26—C21—C22—C23	3.5 (7)
C15—N4—N5—N6	174.3 (3)	N6—C21—C22—C23	−177.6 (5)
Cd1—N4—N5—N6	7.2 (4)	O4—C22—C23—C24	177.1 (5)
N4—N5—N6—C21	179.5 (3)	C21—C22—C23—C24	−2.2 (8)
C13—O1—C1—C6	14.6 (7)	C22—C23—C24—C25	−0.2 (9)
Cd1—O1—C1—C6	166.3 (3)	C23—C24—C25—C26	1.3 (8)
C13—O1—C1—C2	−166.3 (5)	C22—C21—C26—C25	−2.5 (7)
Cd1—O1—C1—C2	−14.5 (4)	N6—C21—C26—C25	178.7 (4)
N2—N1—C2—C3	21.6 (6)	C24—C25—C26—C21	0.1 (7)

### Hydrogen-bond geometry (Å, °)

**Table:**

*D*—H···*A*	*D*—H	H···*A*	*D*···*A*	*D*—H···*A*
O5—H5O···N3	0.91	1.88	2.710 (6)	150
O6—H6O···O4	0.86	1.92	2.739 (5)	157
C23—H23A···Cg1^i^	0.95	2.82	3.655 (6)	147

Symmetry codes: (i) *x*+1, *y*, *z*.
